# Healthcare utilization and cost trajectories post-stroke: role of caregiver and stroke factors

**DOI:** 10.1186/s12913-018-3696-3

**Published:** 2018-11-22

**Authors:** Shilpa Tyagi, Gerald Choon-Huat Koh, Luo Nan, Kelvin Bryan Tan, Helen Hoenig, David B. Matchar, Joanne Yoong, Eric A. Finkelstein, Kim En Lee, N. Venketasubramanian, Edward Menon, Kin Ming Chan, Deidre Anne De Silva, Philip Yap, Boon Yeow Tan, Effie Chew, Sherry H. Young, Yee Sien Ng, Tian Ming Tu, Yan Hoon Ang, Keng Hee Kong, Rajinder Singh, Reshma A. Merchant, Hui Meng Chang, Tseng Tsai Yeo, Chou Ning, Angela Cheong, Yu Li Ng, Chuen Seng Tan

**Affiliations:** 1 Saw Swee Hock School of Public Health, National University of Singapore, 12 Science Drive 2, #10-01, Singapore, 117549 Singapore; 2 Policy Research & Economics Office, Ministry of Health, Singapore, Singapore; 3 Physical Medicine and Rehabilitation Service, Durham VA Medical Centre, Durham, USA; 4 Program in Health Services and Systems Research, Duke-NUS Graduate Medical School, Singapore, Singapore; 5 Lee Kim En Neurology Pte Ltd, Singapore, Singapore; 6 Raffles Neuroscience Centre, Raffles Hospital, Singapore, Singapore; 7 St. Andrew’s Community Hospital, Singapore, Singapore; 8 Mount Alvernia Hospital, Singapore, Singapore; 9 National Neuroscience Institute, Singapore General Hospital campus, Singapore, Singapore; 10 Geriatric Medicine, Khoo Teck Puat Hospital, Singapore, Singapore; 11 St. Luke’s Hospital, Singapore, Singapore; 12 Department of Rehabilitation Medicine, National University Hospital, Singapore, Singapore; 13 Department of Rehabilitation Medicine, Changi General Hospital, Singapore, Singapore; 14 Department of Rehabilitation Medicine, Singapore General Hospital, Singapore, Singapore; 15 Department of Neurology, National Neuroscience Institute, Neurology, Tan Tock Seng Hospital, Singapore, Singapore; 16 Department of Rehabilitation Medicine, Tan Tock Seng Hospital, Singapore, Singapore; 17 Department of Medicine, Yong Loo Lin School of Medicine, National University of Singapore, Singapore, Singapore; 18 Department of Neurosurgery, National University Hospital, Singapore, Singapore

**Keywords:** Stroke, Caregivers, Health services, Healthcare costs, Hospitalization

## Abstract

**Background:**

It is essential to study post-stroke healthcare utilization trajectories from a stroke patient caregiver dyadic perspective to improve healthcare delivery, practices and eventually improve long-term outcomes for stroke patients. However, literature addressing this area is currently limited. Addressing this gap, our study described the trajectory of healthcare service utilization by stroke patients and associated costs over 1-year post-stroke and examined the association with caregiver identity and clinical stroke factors.

**Methods:**

Patient and caregiver variables were obtained from a prospective cohort, while healthcare data was obtained from the national claims database. Generalized estimating equation approach was used to get the population average estimates of healthcare utilization and cost trend across 4 quarters post-stroke.

**Results:**

Five hundred ninety-two stroke patient and caregiver dyads were available for current analysis. The highest utilization occurred in the first quarter post-stroke across all service types and decreased with time. The incidence rate ratio (IRR) of hospitalization decreased by 51, 40, 11 and 1% for patients having spouse, sibling, child and others as caregivers respectively when compared with not having a caregiver (*p* = 0.017). Disability level modified the specialist outpatient clinic usage trajectory with increasing difference between mildly and severely disabled sub-groups across quarters. Stroke type and severity modified the primary care cost trajectory with expected cost estimates differing across second to fourth quarters for moderately-severe ischemic (IRR: 1.67, 1.74, 1.64; *p* = 0.003), moderately-severe non-ischemic (IRR: 1.61, 3.15, 2.44; *p* = 0.001) and severe non-ischemic (IRR: 2.18, 4.92, 4.77; *p* = 0.032) subgroups respectively, compared to first quarter.

**Conclusion:**

Highlighting the quarterly variations, we reported distinct utilization trajectories across subgroups based on clinical characteristics. Caregiver availability reducing hospitalization supports revisiting caregiver’s role as potential hidden workforce, incentivizing their efforts by designing socially inclusive bundled payment models for post-acute stroke care and adopting family-centered clinical care practices.

**Electronic supplementary material:**

The online version of this article (10.1186/s12913-018-3696-3) contains supplementary material, which is available to authorized users.

## Background

Stroke is a catastrophic illness with long term sequelae of disability, dependency and other emotional, psychosocial and financial issues. Globally, about 16 million first stroke cases occur annually with 5.7 million deaths [1]. Over the past two decades, the absolute numbers of stroke cases and the DALYs lost due to it have been increasing [2]. In Singapore, stroke was the 10th most common cause of hospitalization in 2012 and was one of the top five causes of death in 2013, accounting for 8.9% of the total mortality [3, 4]. Stroke not only causes pain and suffering to the patients and their families, but also taxes the economies worldwide, and is accountable for almost 4% of direct healthcare costs in developed nations [5].

A recent study in Singapore reported significant economic strain attributable to direct medical cost of stroke, with mean annual cost being SGD12,473.70. More than 90% of this amount was accounted by inpatient service use, about 5% by outpatient services and less than 2% by emergency services [6]. From a public health perspective, this holds particular significance in Singapore context with rapidly aging population, declining citizen old age support ratio and decreasing fertility rate. Singapore would be home to 900,000 elderly citizens by 2030 and consequently there would be an increase not only in the prevalence of stroke, but also healthcare services utilization (HSU) by both the patient and the caregiver and the associated costs to the healthcare system and society [7, 8].

Furthermore, advancements in medical management strategies have led to decreased stroke attributable deaths, leading to a greater number of survivors, with half of them having residual physical disabilities and cognitive deficits [9, 10] resulting in increased use of healthcare resources.

Considering the economic strain attributable to stroke, researchers have reported the magnitude of stroke associated costs across different settings. Adopting a societal perspective, Bastida and colleagues reported the stroke related average cost per person in Spain to be €17,618 over 1 year. This cost was inclusive of medical costs, informal care costs and productivity loss related costs [11]. Another study focusing on direct costs reported mean hospital related costs per person to be €3624.9 [12]. Within USA, Taylor and colleagues reported lifetime cost per ischemic stroke survivor to be $90,981 [13]. A recent systematic review synthesized the costs associated with post stroke care and reported mean cost per patient month after stroke to be $1515 for studies including both inpatient and outpatient care services and $820 for studies focusing only on outpatient care services. Moreover, they concluded USA to have the highest post-stroke care associated costs, followed by Denmark, Netherlands and Norway. Italy, UK and Germany had one of the lowest post-stroke care associated costs [14].

Temporal trends of index-stroke hospitalization [15], recurrent stroke admissions [16, 17], inpatient rehabilitation utilization [18] and associated risk factors [19] have been described previously, however papers studying post-stroke care trajectories are sparse [20]. Focusing on the index stroke admission, researchers explored the temporal trends over a decade in UK setting and further explored the influence of patient socio-demographic (race and age) and functional characteristics on inpatient acute care and indicators of provision of rehabilitation [21]. Studying aggregated summary estimates at episode level does not provide information on utilization at individual level which is necessary to improve healthcare delivery, reduce unnecessary consumption and improve outcomes for stroke patients. Past work supports significance of first year post-stroke from financial perspective with associated higher consumption and costs [6, 22].

A prospective study in Sweden examined the utilization of different healthcare resources and rehabilitation outcomes over 1-year period in 258 stroke survivors. They reported age of stroke patient and stroke severity as significant covariates [23].

With population of stroke patients (*N* = 674) who underwent inpatient rehabilitation in US setting, Ottenbacher and colleagues examined the prevalence of hospitalizations after index stroke over a 3-month time period. They also studied the association of socio-demographic and clinical covariates with rehospitalization and reported functional dependence and perceived social support to be significantly associated with rehospitalizations [24].

Another study focusing on stroke survivors discharged home in US, was among the select few internationally, which explored the role of caregivers in healthcare seeking post-stroke and reported having a co-residing caregiver being associated with variations in HSU [25]. In concordance with these findings, Roth et al. studied association of co-residing caregiver with HSU after stroke and reported having a co-residing caregiver was associated with reduced length of hospitalization, fewer emergency department visits and fewer primary care visits [26].

Among post-stroke HSU studies done in an Asian setting [6, 27–31], the focus varies with some describing only healthcare services [27, 28], some quantifying financial burden [6, 31] whereas others focusing on both aspects. Only one focused on a post-stroke year-long period [30] while the rest focused on either the index episode [28, 31–33] or duration of 3 months or less after stroke. Commonly described covariates were patient related [12, 23, 24, 31, 34–37] with little focus on caregiver covariates. Addressing above gaps, the aim of our study was to describe the trajectory of HSU by stroke patients and associated costs over 1-year post-stroke and examine the role of caregiver identity. The objectives were: (i) to describe the HSU and cost trajectory across four post-stroke quarters for inpatient, emergency department (ED), specialist outpatient clinic (SOC) and primary care (PC) services, (ii) to study the association of HSU with caregiver identity and patient covariates, (iii) to explore if patient covariates modified the utilization and cost trajectory.

## Methods

We hypothesized: (i) the use and associated costs of acute (inpatient and ED) services would decrease over time as patient’s condition would stabilize, while the use of outpatient (PC and SOC) services would increase, (ii) social support (caregiver availability and type) would decrease the utilization of acute services and increase the utilization of outpatient services, and (vi) the strength of association of patient covariates with acute and outpatient services will change across time.

### Background and setting

Singapore has a mixed healthcare delivery system where public, private and non-profit healthcare institutions deliver inpatient and ED services, intermediate and long-term care (ILTC) as well as specialist and primary ambulatory care. The public sector is the dominant provider of inpatient, ED and specialist outpatient services in tertiary hospitals accounting for more than 80% of market share in these services. The ILTC sector comprises of step-down care, nursing homes and hospice facilities. The sector also includes day services like day rehabilitation, home care services and others. Ministry of Health is the overarching regulatory body responsible for provision and regulation of healthcare services. Stroke patients present with acute symptoms at ED of any of the 5 public tertiary hospitals and are subsequently stabilized and transferred to ward. They undergo rehabilitation and following discharge, are referred to outpatient services to continue care in the community.

### Singapore stroke study

This was a prospective study in Singapore with recruitment period extending from December 2010 to September 2013. Stroke patients and their caregivers were recruited from all five tertiary hospitals of Singapore during that period, which ensured representativeness of our sample. Stroke patients were eligible if they were: (i) Singaporean or permanent resident, more than 40 years old and residing in Singapore for the next 1 year, (ii) stroke must be: recent diagnosis (i.e. stroke symptoms occurring within 4 weeks prior to admission) with diagnosis made by clinician and/or supported by brain imaging (CT or MRI) and (iii) not globally aphasic. A caregiver could be an immediate or extended family member or friend, more than 21 years (the legal definition of adult in Singapore), providing care or assistance of any kind and taking responsibility for the patient, as recognized by the patient and not fully paid for caregiving.

Within each of these tertiary hospitals, on-site research nurses reviewed the list of admitted stroke patients on a daily basis to screen eligible subjects and invited them to participate in the study. Written informed consent was obtained from all participants after explaining the study and procedures involved in the language they understood. They were informed that at any point during the follow-up period of 1 year, they can withdraw from the study, if they wished. Participants (including both the stroke patients and their caregivers) were interviewed via face-to-face interviews at baseline, 3-month and 12-month time points and only caregivers were interviewed via telephone interviews at 6-month and 9-month time points. Self-reported data was collected broadly under health, social and financial domains. To ensure good compliance, reminders were sent 1 week prior to the scheduled follow-up in the form of mails, phone calls or text messages. Interviews were scheduled over weekends or evenings of weekdays for participant’s convenience and adherence. Multiple attempts were made to contact participants before categorizing them as lost to follow-up. Training of first generation research assistants was conducted by main investigators, covering the content and appropriate method of data collection. The recordings of these training sessions were used to train subsequent research assistants to standardize data collection process. We pilot tested our survey with 40 participants from two of the five sites and made necessary amendments and finalized the survey forms.

For current study, while outcome variables were extracted from the national claims record, independent variables were taken from the survey conducted in Singapore Stroke Study at recruitment. National claims record is a nation-wide database of healthcare services utilization and associated costs maintained at the Ministry of Health, Singapore. All Singapore citizens and permanent residents are assigned a unique identification number which is used in almost all administrative contexts, including healthcare utilization. Linkage across multiple databases can be successfully accomplished using this identifier. For current study, adopting this approach the match rate was more than 95%.

Our main independent variable was quarter, a categorical variable representing each 3-month period post-stroke ranging from quarter 1 (Q1) to quarter 4 (Q4). Other covariates, collected from the study were socio-demographic, clinical, functional and caregiver related. The variables included under the socio-demographic group were: patient’s age, gender, ethnicity, religion, marital status, comorbid status and ward class (proxy for socio-economic status). Ward class referred to type of hospital ward stroke patient stayed in during the index stroke hospitalization. In Singapore, based on fulfilling means tested eligibility criteria, patients can be admitted in wards ranging from A, B1, B2 and C with different level of government subsidy. We binarized this ward class variable into two categories of subsidized and non-subsidized ward class.

Those under clinical group were: stroke type, recurrence and severity measured on the National Institute of Health Stroke Scale (NIHSS). Functional status was assessed using two scales, Modified Rankin Scale (mRS) and Barthel Index (BI) [38, 39]. No collinearity was observed between BI and mRS and both were included as covariates. Patients’ cognitive status was recorded using mini-mental state examination (MMSE) [40], while depression was assessed using Center for Epidemiological Studies Depression scale (CES-D-11 scale) [41]. Primary caregiver was an immediate or extended family member or friend, designated by the patient as the person providing physical care and their responses were categorized as none, spouse, child, sibling or others (includes distant relatives and friends).

HSU and associated cost data were extracted from the national claims record, over 3-monthly periods or quarters to study the trajectory and associated variation in HSU and associated costs over one-year post-stroke. Our HSU outcome variables were counts of visits per quarter for inpatient and ED service (excluding the index-stroke event), SOC and PC visits. We extracted total costs (inclusive of subsidies and out-of-pocket components) for inpatient, ED and PC visits.

### Analysis

Liang and Zeger [42] introduced the Generalized Estimating Equation (GEE) approach for analyzing data with repeated measures for Generalized Linear Models which provides population average estimates. For the count data, we chose poisson or negative binomial distribution with a log link function pending on whether there was evidence of over-dispersion [43]. Gamma distribution with a log link was used for cost data. We specified working correlation matrix corresponding to an autoregressive order 1 correlation structure and used the Huber and White sandwich estimator to obtain robust variance estimates even if the working correlation matrix is misspecified [42, 44, 45].

A simple model was run initially to obtain the unadjusted HSU or associated cost trend across the four quarters post-stroke (and is referred as Model 1). Covariates with *p*-value less than 0.1 in the bivariate analysis were considered in the multiple regression models. Backward variable selection was performed to determine the most parsimonious main effects model by removing the most insignificant variable (except for quarters, and age, gender and ethnicity of patient) until only the variables with the p-value < 0.05 remained in the model (and is referred as Model 2). With this adjusted model or Model 2, we further added interaction terms between the quarter variable and time-invariant covariates found significant in Model 2 using forward variable selection approach until the remaining interaction terms that did not enter the model had p-value > 0.05 (referred as Model 3). Model 2 and 3 were adjusted for patient’s demographic factors (age, gender and ethnicity). Significance level was set at 5%. All statistical analysis was performed in Stata 14.

## Results

Five hundred ninety-two stroke patients were available for current analysis, after matching across both databases and exclusion of patients with deaths within the follow up period. For participant flowchart, please refer Additional file 1. As shown in Table 1, majority of the participants were less than 65 years old, Chinese, married males. About 88% had ischemic stroke and 61, 35 and 4% were mild, moderate and severe strokes respectively. About half of the caregivers providing physical care to the stroke patients were spouses (50.8%), followed by sibling (26%), child (6%) and others (6%) and about 11% of the current cohort reported as having no caregiver.

**Table 1:** Baseline socio-demographic and clinical characteristics

	N^a^ (%)
Age	
< 65 years	367 (62.0)
> = 65 years	225 (38.0)
Gender	
Male	393 (66.4)
Female	199 (33.6)
Ethnicity	
Chinese	402 (67.9)
Non-Chinese	190 (32.1)
Religion	
Religion	542 (91.7)
No Religion	49 (8.3)
Marital status	
Married	413 (69.8)
Single	179 (30.2)
Comorbid conditions present	
No	66 (11.2)
Yes	526 (88.8)
Ward class	
Unsubsidized	50 (8.5)
Subsidized	542 (91.5)
Stroke Type	
Ischemic	518 (87.8)
Non-ischemic	72 (12.2)
National Institute of Health Stroke Scale	
Mild (0–4)	339 (60.5)
Moderately severe (5–14)	196 (35.0)
Severe (15–24)	25 (4.5)
Barthel Index	
Independence (100)	130 (24.7)
Slight Dependence (91–99)	82 (15.6)
Moderate Dependence (61–90)	156 (29.7)
Severe Dependence (21–60)	80 (15.2)
Total Dependence (0–20)	78 (14.8)
Modified Rankin Scale	
No or slight disability (0–2)	255 (43.8)
Moderate or severe disability (3–5)	327 (56.2)
Mini-Mental State Examination	
No cognitive impairment	312 (63.2)
Mild cognitive impairment	121 (24.5)
Severe cognitive impairment	61 (12.3)
Frontal Assessment Battery^b^	
Mean (SD)	14 (3.9)
Centre for Epidemiological Studies Depression Scale^b^	
Mean (SD)	6.5 (5.5)
Discharge to Community Hospital	
Yes	139 (23.5)
No	452 (76.5)
Relationship with caregiver	
None	66 (11.2)
Spouse	299 (50.8)
Child	151 (25.7)
Sibling	34 (5.8)
Others	38 (6.5)

^a^All numbers may not add up to total because of missing data

^b^Missing values: Frontal Assessment Battery (70); Centre for Epidemiological Studies Depression Scale (45)

### Healthcare service utilization and associated costs post-stroke (Table 2)

**Fig. 1:**
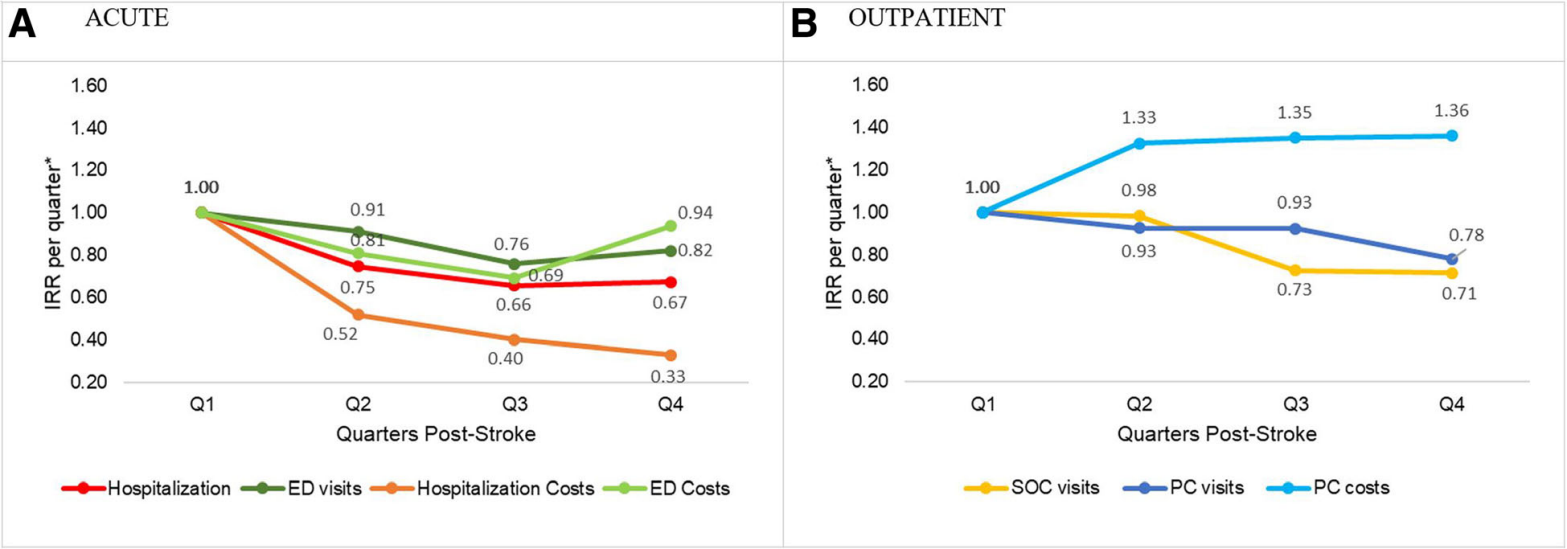
Acute and outpatient healthcare service utilization and associated costs across 4 quarters post-stroke. Estimates are taken from Model 2 with following variables in final model. *Service utilization model.* Acute inpatient service: patient age, gender, ethnicity, caregiver identity, comorbid status; Acute ED service: patient age, gender, ethnicity, caregiver identity, comorbid status, religion; Outpatient SOC service: patient age, gender, ethnicity, stroke disability (measured on modified rankin scale), comorbid status; Outpatient PC service: patient age, gender, ethnicity, stroke type, stroke severity, ward class, comorbid status. *Cost model.* Acute inpatient cost: age, gender, ethnicity, caregiver identity, comorbid status, marital status, recurrent stroke; Acute ED cost: age, gender, ethnicity, caregiver identity, comorbid status; Outpatient PC cost: age, gender, ethnicity, stroke type, stroke severity, comorbid status. **a** Inpatient and emergency department service utilization and costs (ACUTE), **b** Specialist outpatient clinic and primary care utilization and costs (OUTPATIENT). Abbreviations: IRR = incidence rate ratio; ED = emergency department; SOC = specialist outpatient clinic; PC = primary care. *: For Hospitalization/ED (or PC) cost, the y-axis is the ratio of expected cost from Q2 to Q4 to the reference quarter (Q1) respectively

Compared to first quarter post-stroke, the unadjusted incidence rate ratio (IRR) of acute hospitalization in subsequent three quarters was 0.74 (95% CI: 0.54, 1.02), 0.65 (95% CI: 0.48, 0.88) and 0.67 (95% CI: 0.47, 0.96) respectively. The IRR estimates remained almost unchanged after adjusting for covariates. A decreasing trend of acute hospitalization associated cost was observed with adjusted exponentiated cost estimates from Q2 to Q4 being 0.52 (95% CI: 0.32, 0.83), 0.40 (95% CI: 0.23, 0.68) and 0.33 (95% CI: 0.20, 0.54) when compared with Q1 suggesting expected acute hospitalization associated cost decreased by 48, 60 and 67% for Q2, Q3 and Q4 respectively when contrasted with Q1. Both adjusted acute hospitalization and associated cost post-stroke showed a statistically significant linear trend effect that decreased with time since stroke, with greater decrease in the cost compared to number of visits (Fig. 1). Compared to first quarter, the adjusted IRR of ED service use in subsequent quarters was 0.91 (95% CI: 0.70, 1.19), 0.76 (95% CI: 0.58, 0.99) and 0.82 (95% CI: 0.57, 1.17) respectively. Both ED visit and associated cost showed a decreasing trend till third quarter post-stroke, but the linear trend effect was not statistically significant (Fig. 1). Compared to first quarter post-stroke, the unadjusted IRR of SOC service use in subsequent three quarters were 1.00 (95% CI: 0.89, 1.13), 0.75 (95% CI: 0.67, 0.84) and 0.74 (95% CI: 0.65, 0.85) respectively. After adjustment, the IRR of SOC service use had a similar significant linear trend with utilization still being the highest in the first 6 months and decreasing subsequently (Fig. 1). PC service utilization had a significant overall trend where the highest utilization was in the quarter immediately post-stroke and then decreased subsequently with adjusted IRR of PC visits per quarter being 0.93 (95% CI: 0.81, 1.06), 0.93 (95% CI: 0.79, 1.09) and 0.78 (95% CI: 0.67, 0.90) respectively in Q2 to Q4 when compared with Q1. The PC service associated cost showed a significant linear trend that had an opposite trend to PC visits with adjusted, exponentiated cost estimates in Q2, Q3 and Q4 being 1.33 (95% CI: 1.14, 1.54), 1.35 (95% CI: 1.15, 1.59) and 1.36 (95% CI: 1.15, 1.62) when compared with Q1 suggesting expected PC associated cost increased by 33, 35 and 36% for Q2, Q3 and Q4 respectively when contrasted with Q1 (Fig. 1). While both cost and service usage decreased across post-stroke quarters for inpatient and ED services, the service usage decreased, and cost increased across post-stroke quarters for PC services.

**Table 2:** Trend estimates for post-stroke healthcare service utilization and costs

	Q1	Q2	Q3	Q4	*p***-**value
Healthcare Service Used		IRR (95% CI)	IRR (95% CI)	IRR (95% CI)	
Acute (Inpatient) service					
Model 1	Ref	0.74 (0.54, 1.02)	0.65 (0.48, 0.88)	0.67 (0.47, 0.96)	0.020
Model 2	Ref	0.75 (0.54, 1.03)	0.66 (0.48, 0.89)	0.67 (0.47, 0.96)	0.020
Acute (ED) service					
Model 1	Ref	0.90 (0.69, 1.18)	0.75 (0.58, 0.99)	0.82 (0.58, 1.17)	0.181
Model 2	Ref	0.91 (0.70, 1.19)	0.76 (0.58, 0.99)	0.82 (0.57, 1.17)	0.182
Outpatient (SOC) service					
Model 1	Ref	1.00 (0.89, 1.13)	0.75 (0.67, 0.84)	0.74 (0.65, 0.85)	< 0.001
Model 2	Ref	0.98 (0.87, 1.11)	0.73 (0.65, 0.81)	0.71 (0.63, 0.81)	< 0.001
Outpatient (PC) service					
Model 1	Ref	0.91 (0.79, 1.04)	0.89 (0.76, 1.04)	0.77 (0.67, 0.89)	0.001
Model 2	Ref	0.93 (0.81, 1.06)	0.93 (0.79, 1.09)	0.78 (0.67, 0.90)	0.002
Healthcare Costs		Exp(β) (95% CI)	Exp(β) (95% CI)	Exp(β) (95% CI)	
Acute (Inpatient) costs					
Model 1	Ref	0.65 (0.43, 0.98)	0.66 (0.40, 1.09)	0.50 (0.31, 0.79)	0.006
Model 2	Ref	0.52 (0.32, 0.83)	0.40 (0.23, 0.68)	0.33 (0.20, 0.54)	< 0.001
Acute (ED) costs					
Model 1	Ref	0.86 (0.62, 1.19)	0.74 (0.53, 1.03)	0.95 (0.62, 1.45)	0.643
Model 2	Ref	0.81 (0.58, 1.13)	0.69 (0.49, 0.97)	0.94 (0.66, 1.34)	0.538
Outpatient (PC) costs					
Model 1	Ref	1.28 (1.11, 1.48)	1.23 (1.06, 1.43)	1.30 (1.11, 1.53)	0.004
Model 2	Ref	1.33 (1.14, 1.54)	1.35 (1.15, 1.59)	1.36 (1.15, 1.62)	0.001

Model 1 has only quarter in the model; Model 2 has the additional covariates in the model (see below for details)

*Service utilization model.* Acute inpatient service: patient age, gender, ethnicity, caregiver identity, comorbid status; Acute ED service: patient age, gender, ethnicity, caregiver identity, comorbid status, religion; Outpatient SOC service: patient age, gender, ethnicity, stroke disability (measured on modified rankin scale), comorbid status; Outpatient PC service: patient age, gender, ethnicity, stroke type, stroke severity, ward class, comorbid status

*Cost model.* Acute inpatient cost: age, gender, ethnicity, caregiver identity, comorbid status, marital status, recurrent stroke; Acute ED cost: age, gender, ethnicity, caregiver identity, comorbid status; Outpatient PC cost: age, gender, ethnicity, stroke type, stroke severity, comorbid status

*Abbreviations*: *Ref* reference category, *IRR* incidence rate ratio, *CI* confidence interval, *ED* emergency department, *SOC* specialist outpatient clinic, *PC* primary care, *Exp(β)* exponentiated beta coefficient corresponds to the ratio of expected cost from Q2 to Q4 to the reference quarter respectively

### Caregiver identity and use of inpatient services and associated costs

**Fig. 2:**
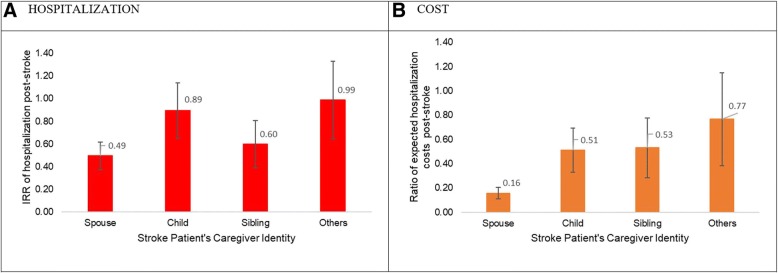
Hospitalization and associated costs by caregiver identity. Estimates are taken from Model 2 with following variables in final model. *Service utilization model.* Acute inpatient service: patient age, gender, ethnicity, caregiver identity, comorbid status. *Cost model.* Acute inpatient cost: age, gender, ethnicity, caregiver identity, comorbid status, marital status, recurrent stroke. Reference group for caregiver identity variable is stroke patients with no caregiver. **a** Incidence risk ratio of hospitalization by caregiver identity (HOSPITALIZATION), **b** Multiplier of hospitalization associated costs by caregiver identity (COST). Abbreviation: IRR = incidence rate ratio. *: For hospitalization cost, the y-axis is the ratio of expected cost from Q2 to Q4 to the reference quarter (Q1) respectively

Caregiver identity was associated with acute inpatient and ED visits and their associated costs only. The IRR of acute hospitalization decreased by 51, 40, 11 and 1% for patients having spousal, sibling, child and others as caregivers respectively when compared with those having no caregiver (*p* = 0.017). Like hospitalization, acute hospitalization related costs for those with any related caregiver were lower than those with no caregiver. Having a spousal caregiver was associated with the lowest acute hospitalization costs with exponentiated cost estimate being 0.16 (95% CI: 0.09, 0.29) when compared with no caregivers suggesting expected hospitalization cost decreased by 84% when contrasted with no caregivers (*p* < 0.001) (Fig. 2).

### Disability post-stroke and use of SOC services

**Fig. 3:**
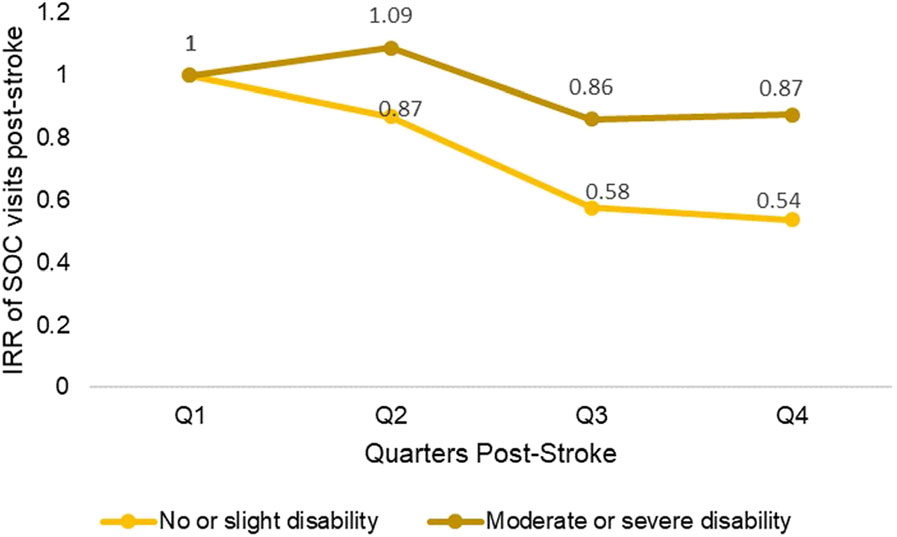
Specialist outpatient visits across 4 quarters post-stroke by disability sub-groups. Estimates taken from Model 3 with following variables in the final model. Service utilization model. Outpatient SOC service: patient age, gender, ethnicity, stroke disability (measured on modified rankin scale), comorbid status. Disability measured using Modified Rankin Scale (mRS): mRS score of 0 to 2 = no or slight disability group, mRS score of 3 to 5 = moderate or severe disability. Abbreviation: IRR = incidence rate ratio; SOC = specialist outpatient clinic

The interaction term between quarter and disability (measured on mRS) was statistically significant (*p* < 0.001) in SOC service utilization model suggesting the utilization trend was different among varying levels of disability in stroke patients. Though the SOC utilization trend was decreasing across post-stroke quarters for both no or slightly disabled (mRS: 0–2) and moderate or severely disabled groups (mRS: 3–5), the decrease was steeper for the group with no or slight disability (Fig. 3) where the linear trend of SOC utilization across 4 quarters post-stroke in both groups were statistically significant (Table 3).

**Table 3:** Trend estimates for post-stroke healthcare service utilization and costs by sub-groups

	Q1	Q2	Q3	Q4	*p***-**value
Outpatient (SOC) Service^a^	IRR	IRR (95% CI)	IRR (95% CI)	IRR (95% CI)	
Disability (mRS)					
No or slight disability (0–2)	Ref	0.87 (0.68, 1.10)	0.58 (0.50, 0.67)	0.54 (0.46, 0.63)	< 0.001
Moderate or severe disability (3–5)	Ref	1.09 (0.96, 1.23)	0.86 (0.74, 1.00)	0.87 (0.73, 1.05)	0.035
Outpatient (PC) Costs^a^	Exp(β)	Exp(β) (95% CI)	Exp(β) (95% CI)	Exp(β) (95% CI)	
Ischemic stroke					
Mild (0–4)	Ref	1.15 (0.97, 1.35)	1.02 (0.85, 1.21)	1.10 (0.91, 1.33)	0.577
Moderately severe (5–14)	Ref	1.67 (1.22, 2.30)	1.74 (1.30, 2.33)	1.64 (1.18, 2.29)	0.003
Severe (15–24)	Ref	2.27 (0.56, 9.15)	2.72 (0.69, 10.74)	3.20 (0.73, 13.97)	0.102
Non-Ischemic stroke					
Mild (0–4)	Ref	1.10 (0.64, 1.91)	1.84 (1.01, 3.35)	1.64 (0.85, 3.15)	0.067
Moderately severe (5–14)	Ref	1.61 (0.97, 2.67)	3.15 (1.76, 5.65)	2.44 (1.33, 4.48)	0.001
Severe (15–24)	Ref	2.18 (0.47, 10.14)	4.92 (0.86, 28.18)	4.77 (0.97, 23.41)	0.032

*Abbreviations*: *Ref* reference category, *IRR* incidence rate ratio, *CI* confidence interval, *SOC* specialist outpatient clinic, *PC* primary care, *Exp(β)* exponentiated beta coefficient corresponds to the ratio of expected cost from Q2 to Q4 to the reference quarter respectively

^a^Effect estimates based on Model 3: SOC Service included interaction between mRS and quarter (*p* < 0.001) term; PC Cost included interaction between stroke type and quarter (*p* = 0.017) and between severity and quarter (*p* = 0.039) terms

### Stroke type and severity, and primary care associated costs

**Fig. 4:**
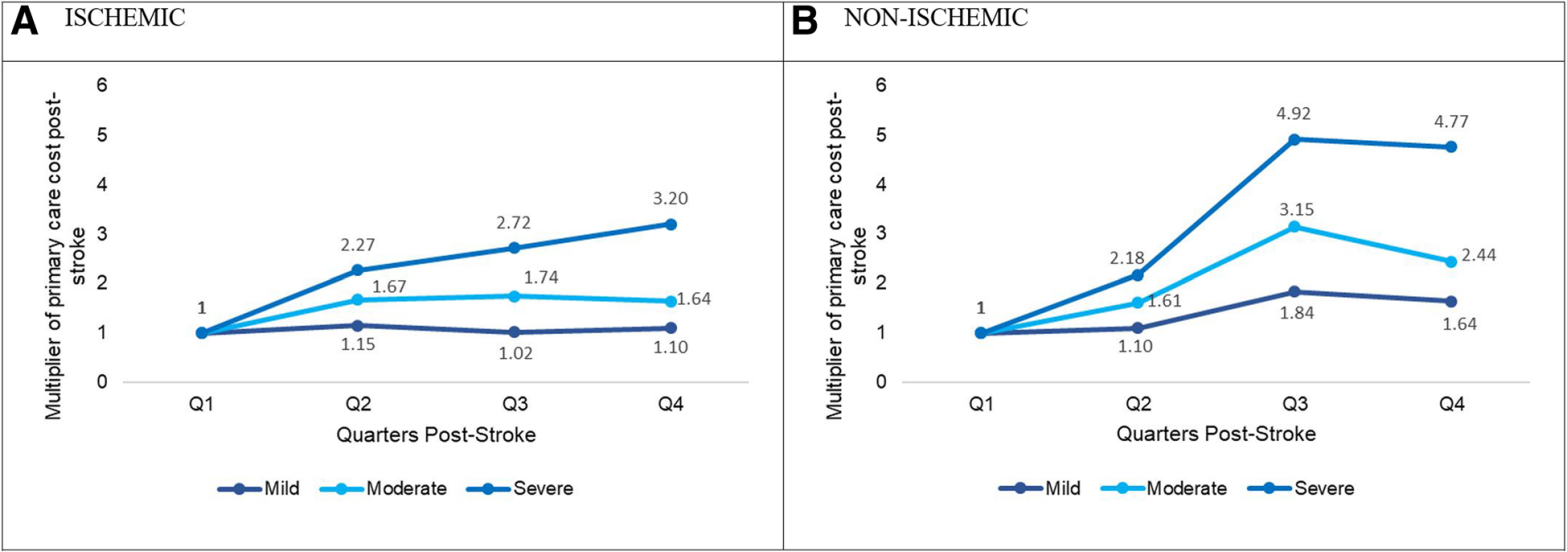
Primary care costs across 4 quarters post-stroke by stroke type and severity. Estimates taken from Model 3 with following variables in the final model. Cost model. Outpatient PC cost: age, gender, ethnicity, stroke type, stroke severity, comorbid status. **a** Ratio of expected primary care costs by stroke severity in ischemic stroke sub-group (ISCHEMIC), **b** Ratio of expected primary care costs by stroke severity in non-ischemic stroke sub-group (NON-ISCHEMIC). Stroke severity measured using National Institute of Health Stroke Scale (NIHSS): mild = 0 to 4, moderately severe = 5 to 14, severe = 15 to 24. Abbreviations: Mild = mild stroke; Moderate = moderately severe stroke; Severe = severe stroke *: For primary care cost, the y-axis is the ratio of expected cost from Q2 to Q4 to the reference quarter (Q1) respectively

The PC costs across post-stroke quarters varied by stroke type and severity. Figure 4(a) illustrates the PC cost trend having an increasingly pronounced positive linear trend as we progressed from mild, moderately severe to severe ischemic stroke subgroups although the linear trend of PC associated cost was statistically significant only for moderately severe ischemic group with borderline significance (*p* < 0.15) for severe ischemic group (Table 3). Figure 4(b) illustrates the PC cost trend for mild, moderately severe and severe non-ischemic stroke subgroups. For all three groups, compared to cost in Q1, there was an increase in Q2, Q3 after which the increase in Q4 was slightly lower than Q3. However, the magnitude of increase across Q2 to Q4, compared to Q1 was increasingly pronounced as stroke severity increased where the linear trend for PC service associated costs was statistically significant for moderately severe and severe non-ischemic groups with borderline significance (*p* < 0.15) for mild non-ischemic group (Table 3). The overall results are summarized in Table 4.

**Table 4:** Summary of findings

Independent Factors	HEALTHCARE SERVICE						
	Utilization				Cost		
	Acute		Outpatient		Acute		Outpatient
	IN	ED	SOC	PC	IN	ED	PC
Quarters (Q1 to Q4)^a^	↓	NS	↓	↓	↓	NS	↑
Caregiver (present vs none)^a^	↓	↓	NA^d^	NA^d^	↓	↓	NA^d^
Stroke factors							
Physical disability (mRS) Severe (vs Mild)	NA^d^	NA^d^	SS^b,c^	NA^d^	NA^d^	NA^d^	NA^d^
Stroke severity (NIHSS) Severe & moderate (vs mild)	NA^d^	NA^d^	NA^d^	SS^a^	NA^d^	NA^d^	SS^b,c^
Stroke type (ischemic vs non-ischemic)	NA^d^	NA^d^	NA^d^	SS^a^	NA^d^	NA^d^	SS^b,c^

*↓* decreasing trend, ↑  increasing trend, *NS* non-significant, *NA* not applicable, *SS* statistically significant, *IN* inpatient, *ED* emergency department, *SOC* specialist outpatient clinic, *PC* primary care, *mRS* modified Rankin Scale, *NIHSS* National Institute of Health Stroke Scale

^a^based on Model 2

^b^based on Model 3

^c^interaction of covariate with the quarter variable in the final model is statistically significant

^d^the covariate did not enter the final model

## Discussion

Our study is the first to establish the association of caregiver identity with acute hospitalization and associated costs post-stroke. Our results supported the hypothesis of presence of caregiver being associated with decreased hospitalization and its associated costs only. Possible explanation is hospitalization episodes being unplanned, could be a proxy of unmet social needs rendered to stroke patient because of the absence of a caregiver. A study done in Taiwan, concluded higher rehospitalization for elderly participants with caregivers reporting support needs [46]. In contrast, PC and SOC services are voluntary services whose utilization may not be affected by caregiver covariate but be more driven by health needs or clinical stroke characteristics.

Chuang et al. [27] explored association of caregiver factors with post-stroke readmission but found disability level, first-stroke, nursing needs and care arrangements post-discharge being significant predictors of rehospitalization within the 30-day observation period instead of caregiver covariates. Possible explanations could be a short observation period (1-month) and sample limited to stroke patients being functionally impaired at discharge. With longer observation period and inclusion of all stroke patients, caregiver factors may achieve greater significance, as observed in our case.

Roth and colleagues reported the role of co-residing caregivers in stroke survivors’ use of healthcare services over a 6-month period in the United States of America and they concluded having a co-residing caregiver was associated with reduced healthcare consumption after index stroke episode [26]. While the literature on association of caregiver characteristics with HSU in stroke population is still in the nascent stage, there is supporting evidence for this association among other population subgroups like elderly or heart failure patients. Prior work involving frail elderly in Italy reported odds of hospitalization being 2.59 times in those living alone as compared to those living with an informal caregiver [47]. Another study involving heart failure patients concluded presence of spouse or legally registered domestic partner to be associated with reduced risk of readmission within 3 months of index hospital admission [48].

Caregivers can also be viewed as constituents of stroke patient’s social support system and researchers have reported social support to be associated with reduced hospitalizations in both stroke and non-stroke patient population. In concordance with our results, a study done in the United States of America on hospital readmissions within 3 months of discharge from inpatient rehabilitation setting after stroke reported social support to be significantly associated with rehospitalizations. Specifically, those stroke patients having lower social support being 2.28 times more likely to be hospitalized as compared to those with high social support [24].

Another point to note is past efforts are limited to one type of caregiver, mainly children of elderly care recipients [49–51]. However, with aging population and decreasing fertility ratio, it is necessary to expand this analysis to different types of caregivers, especially spouse and sibling whose numbers and importance would increase with time. Since our sample comprised of different categories of caregivers, we not only reported on presence of caregiver and HSU but also found that presence of spousal or sibling caregiver was significantly associated with a decrease in acute hospitalization risk and cost where spousal caregiver had the largest drop. The differentiation across caregiver identity is an interesting and novel finding which is worth exploring further in-depth.

Wolff and colleagues studied the elderly population and association of caregiver characteristics with risk of being hospitalized and with delayed discharge. They reported on bivariate analysis a significant association between caregiver identity and hospitalization, with lower percentage of spousal caregivers (as compared to child and others) in the ever hospitalized group (*p* = 0.032) [52]. Hanaoka and colleagues described association between children caregiver related factors and elderly care recipient’s use of long-term care in Japan. They showed that lower opportunity cost of caregiving and caregivers co-residing with care recipients decreased recipients’ utilization of long term care services [53]. Assuming spousal or sibling caregivers to be older and less likely to be working compared to children caregivers, both would have lower opportunity costs of caregiving and this might explain their association with lower use of acute healthcare services. A recent qualitative study, reported a better match between caregiver’s capacity and care recipient’s needs would enable better adaption to the new role, better coping, less strain and improved patient outcomes. Moreover, they mentioned protective factors exhibited by some caregivers, such as, “self-awareness” (know own physical, emotional limitations) and “self-advocacy” (not hesitating to ask for help) which help them cope well with the new role and potentially would lead to the dyad’s well-being [54]. Based on our results, we propose that these findings may present differently in different caregiver types, with spousal or sibling caregivers potentially being more self-aware and asking for help sooner compared to children caregivers.

Our results have potential implications, since acute hospitalization constitutes the bulk of the financial burden related to stroke. Future research efforts should be directed to study in-depth the role of caregivers in stroke patient’s acute healthcare utilization to garner evidence in support of programs benefitting caregivers. From healthcare financing perspective, bundled payment models are explored to finance such acute and post-acute episodes of care to reduce costs, improve care quality and incentivize vertical integration enabling care coordination [55]. Currently, the scope of such initiatives is limited to developing bundles of care across different clinical settings, mostly limited to acute episodes, with recent interest in post-acute period of care [56, 57]. In light of our finding of family caregivers reducing hospitalization risk post-stroke, studies exploring bundled payment models post-stroke should consider inclusion of social sector or informal caregiving and ascertain the role caregivers can play in such arrangements.

Interestingly, caregiver covariate was only significantly associated with acute service utilization and not with use of outpatient services. Rather index stroke characteristics were associated with utilization and costs of outpatient services. Disability level modified the SOC usage trajectory with SOC usage declining among severely disabled sub-group but less pronounced when compared with mildly disabled group. This finding adds new knowledge to previous literature which reports association of stroke related disability with overall mean level of healthcare service utilization and costs [34, 37, 58]. Potential practical implication will be to focus on severely disabled stroke sub-population during acute or intensive care phase to minimize functional dependence and resultant use of SOC services. Efforts should be aimed at stabilizing the patient and enabling transition in the community.

Index stroke type and severity was significantly associated with PC utilization and further modified the PC cost trajectory across post-stroke quarters (Table 4). For non-ischemic group, the increase in PC cost was observed upto Q3 and a drop in Q4 for all three severity groups, with the linear trend being significant for moderately severe and severe sub-groups. While for ischemic group, the trend was comparatively less pronounced, with the increase in PC cost observed up to Q2, Q3 and Q4 for mild, moderately severe and severe sub-groups. This trend could be explained by the fact that severe cases will take more time to recover, stabilize and their transition to PC setting occurs later compared to milder cases. We also observed that while the PC visit trajectory for the whole stroke cohort showed a decreasing trend over time, the PC visit associated cost trajectory showed an increasing trend over time. Possible explanation of this observation could be in the interaction we observed in the PC cost trajectory model whereby overall more severe stroke sub-group have more steeper increasing cost trend as compared to the less severe stroke sub-groups across subsequent quarters. Thus, it might be the case that more severe stroke patients use more primary care services across subsequent quarters post stroke. Moreover, more severe stroke sub-group will be having more complex healthcare needs and even with overall reduced number of PC visits, this subgroup might be driving up the overall cost associated with use of PC services over time.

Overall, the highest utilization and associated costs (except PC costs) occurred in the first quarter post-stroke across all service types and then decreased with time, though the degree of decrease varied with different services. Similar results were reported previously, with highest hospitalization risk closer to index-event and subsequent decrease with time [37]. Another study described highest outpatient rehabilitation service use in first quarter post-stroke with subsequent drop [35]. Though authors have highlighted the importance of first-year post-stroke from a financial and healthcare perspective [6, 22, 59], by reporting quarterly variations within first post-stroke year, with first 3-months being most crucial, we described distinct utilization trajectories across sub-groups of stroke patients based on clinical characteristics.

Our study has several strengths. We had the advantage of temporality as covariates were collected prior to tracking of healthcare outcomes. While we cannot comment from causality perspective, we can comment on directionality of our findings whereby we are reporting influence of various caregiver characteristics on subsequent healthcare utilization and associated cost trajectories of the stroke patients. Our study had the strength of leveraging on two data sources with one being a national claims record and hence an objective source of healthcare utilization and cost data. With nationwide coverage extending across different healthcare sectors, national claims record provided reliable estimates of utilization and costs data. Moreover, compared to proxy-reported utilization and cost estimates captured in Singapore Stroke Study, estimates taken from national claims record were more accurate being free of reporting bias. Another advantage of using national claims record as a source of outcome variables over the follow-up period was the fact that our reported estimates were not affected by loss to follow-up of study participants. The follow-up rates for study participants were 65.5% at 3 months and 55.8% at 12 months. Our independent variables were obtained from Singapore Stroke Study from the baseline survey and hence using national claims record to capture dependent variables at subsequent time points enabled us to overcome this issue of attrition of study sample over time. Our findings highlight the role of caregiver identity in acute hospitalization and associated costs post-stroke, index stroke characteristics can modify the trajectories of SOC service utilization and PC costs resulting in the identification of different sub-groups within the stroke cohort. Past studies highlight the financial and healthcare implications of first post-stroke year, however we report the quarterly variations within this first-year, with highest utilization occurring within the first 3-months.

Following are some of the limitations of our study. We wanted to quantify the trajectory for stroke patients alive at the end of observation period of 1 year, therefore we excluded those who died during follow-up, which was a relatively small proportion (6%). We conducted sensitivity analysis to compare the baseline characteristics of included and excluded sample of stroke patients with excluded sample mainly comprising of stroke patients who died during follow up. (Refer Additional file 2) Taking both the initial objective to quantify trajectory of stroke survivors and the results from our sensitivity analysis, generalizability of our results will be limited to stroke survivors who are alive 1 year after index stroke.

While national claims record has comprehensive coverage of inpatient, ED and SOC utilization, the PC data coverage was limited to the public sector, since private sector utilization might not be completely captured by national claims record. Potential implication on our findings is we might be underestimating the PC utilization and associated costs. However, since our primary aim was to study the trend or trajectories of healthcare utilization and associated costs across subsequent quarters, and not quantifying the exact consumption of services, our trend estimates will not be affected by this limitation to a significant extent. Moreover, patients who seek care in public sector generally continue to seek care in same setting across subsequent time periods and the same might be applicable for follow up in private setting. Therefore, our reported PC utilization and cost trajectory findings might be applicable to sub-group of stroke patients seeking primary healthcare in public sector. Lastly for the purpose of current study, we incorporated all healthcare utilization and associated costs post index stroke and did not specifically focus on utilization and costs attributable to post-stroke care management plan. Along the same lines, one of the areas we intend to explore in our future publications is the impact of specific comorbidity on healthcare utilization and associated costs.

## Conclusion

We described the trajectory of healthcare service utilization by stroke patients and associated costs over 1-year post-stroke and examined the association of caregiver identity with utilization and costs. Contrary to our hypothesis, we found decreasing utilization and cost trajectories for all services except PC utilization associated costs which showed increasing trend across subsequent quarters post-stroke. Highlighting the quarterly variations within first post-stroke year, with the importance of first 3 months, we reported distinct utilization trajectories across subgroups of stroke patients based on clinical characteristics. Our results supported the hypothesis of social support in the form of caregiver availability and type being associated with decreased hospitalization and its associated costs only. We did not find any significant association of caregiver availability and type with utilization of outpatient services. PC and SOC service utilization and cost trajectories were driven more by clinical and functional stroke variables. We reported distinct SOC utilization trajectories across stroke patient subgroups with varying functional disability (as measured on Modified Rankin Scale) and distinct PC cost trajectories across stroke patient subgroups of different stroke type and varying stroke severity. Our finding of caregiver availability reducing hospitalization supports revisiting caregiver’s role as potential hidden workforce with further incentivizing their efforts by designing socially inclusive bundled payment models for post-acute stroke care in future.

## Additional files

Additional file 1: Study participant flowchart. (PDF 59 kb)

Additional file 2: Sensitivity analysis. Comparison of baseline socio-demographic and clincial characteristics of stroke patients in the main sample and excluded sample. (PDF 36 kb)

## Abbreviations

BI: Barthel Index
CES-D-11 scale: Center for Epidemiological Studies Depression scale
ED: Emergency department
GEE: Generalized Estimating Equation
HSU: Healthcare service utilization
ILTC: Intermediate and long-term care
IRR: Incidence rate ratio
MMSE: Mini-mental state examination
mRS: Modified Rankin Scale
NIHSS: National Institute of Health Stroke Scale
PC: Primary care
Q: Quarter (e.g. Q1 for quarter 1)
SOC: Specialist outpatient clinic
