# Supplementary figures and images for: Healthcare utilization and cost trajectories post-stroke: role of caregiver and stroke factors
Source: BMC Health Serv Res. 2018 Nov 22;18:881. doi: 10.1186/s12913-018-3696-3 (PMC6251229; doi:10.1186/s12913-018-3696-3)

## Study Participant Flowchart

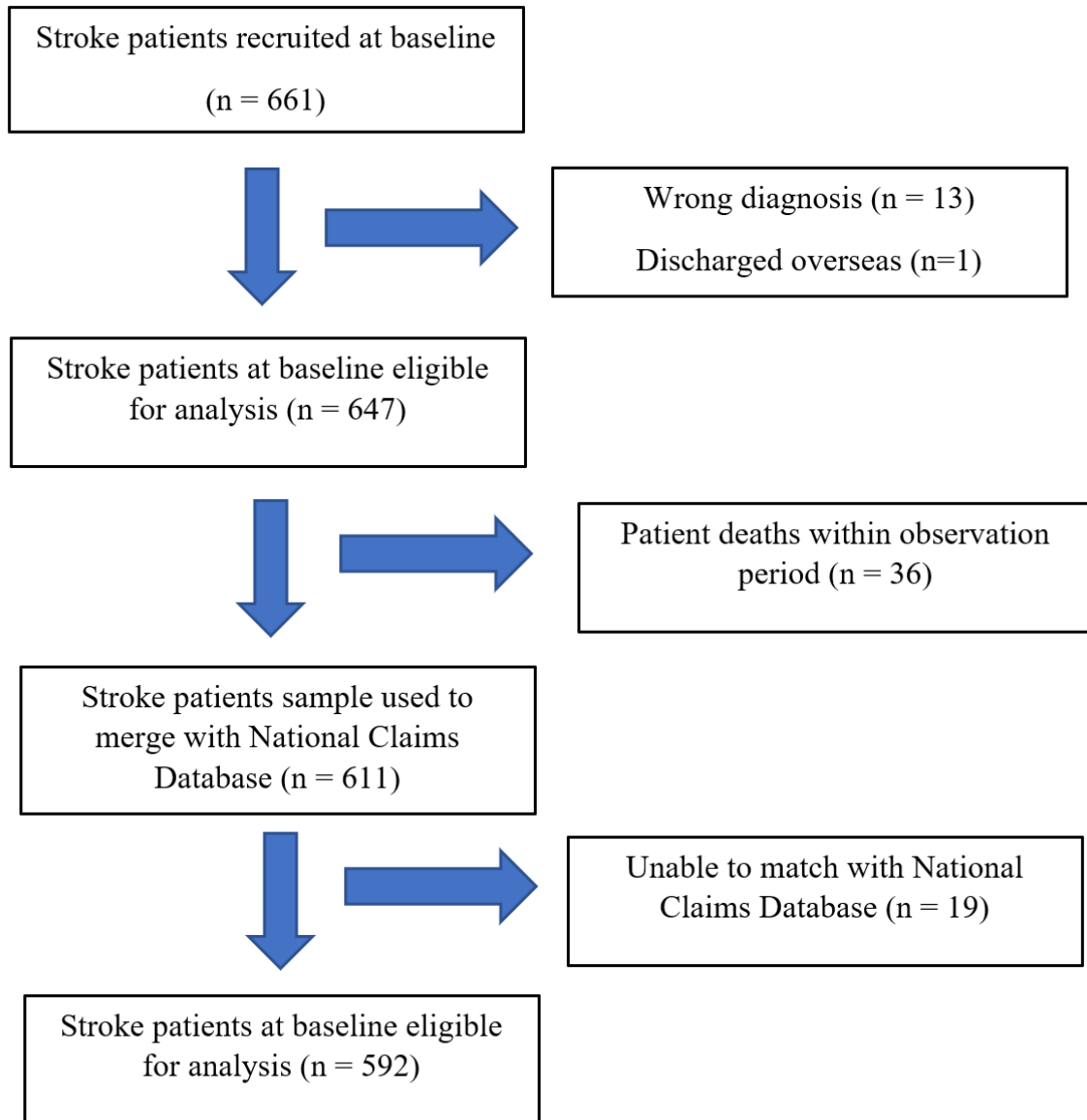

Supplement: Supplementary file 1 — Study participant flowchart. (PDF 59 kb) [file 12913_2018_3696_MOESM1_ESM.pdf]
