# Supplementary material for: Healthcare utilization and cost trajectories post-stroke: role of caregiver and stroke factors
Source: BMC Health Serv Res. 2018 Nov 22;18:881. doi: 10.1186/s12913-018-3696-3 (PMC6251229; doi:10.1186/s12913-018-3696-3)
Supplement: Supplementary file 2 — Sensitivity analysis. Comparison of baseline socio-demographic and clincial characteristics of stroke patients in the main sample and excluded sample. (PDF 36 kb) [file 12913_2018_3696_MOESM2_ESM.pdf]

Sensitivity Analysis

Comparison of baseline socio-demographic and clinical characteristics of stroke patients in the main sample and excluded sample

		Main Sample (592)	Excluded Sample (69)	
		N (%)	N (%)	P-value
Age	< 65 years	367 (62.0)	33 (47.8)	**0.023**
	>= 65 years	225 (38.0)	36 (52.2)	
Gender	Male	393 (66.4)	44 (63.8)	0.664
	Female	199 (33.6)	25 (36.2)	
Ethnicity	Chinese	402 (67.9)	44 (63.8)	0.488
	Non-Chinese	190 (32.1)	25 (36.2)	
Religion	Religion	542 (91.7)	58 (85.3)	0.079
	No Religion	49 (8.3)	10 (14.7)	
Marital status	Married	413 (69.8)	39 (56.5)	**0.025**
	Single	179 (30.2)	30 (43.5)	

Comorbid conditions present	No	66 (11.2)	10 (14.5)	0.410
	Yes	526 (88.8)	59 (85.5)	
Ward class	Unsubsidized	50 (8.5)	2 (4.1)	0.282
	Subsidized	542 (91.5)	47 (95.9)	
Stroke Type	Ischemic	518 (87.8)	58 (87.9)	0.985
	Non-ischemic	72 (12.2)	8 (12.1)	
National Institute of Health Stroke Scale (NIHSS)	Mild (0-4)	339 (60.5)	34 (54.8)	**<0.001**
	Moderately severe (5-14)	196 (35.0)	20 (32.3)	
	Severe (15-24)	25 (4.5)	6 (9.7)	
	Very severe (25-30)	0 (0)	2 (3.2)	
Barthel Index	Independence (100)	130 (24.7)	12 (20.3)	**0.011**
	Slight Dependence (91-99)	82 (15.6)	4 (6.8)	
	Moderate Dependence (61-90)	156 (29.7)	16 (27.1)	
	Severe Dependence (21-60)	80 (15.2)	8 (13.6)	

	Total Dependence (0-20)	78 (14.8)	19 (32.2)	
Modified Rankin Scale	No or slight disability (0-2)	255 (43.8)	21 (31.3)	0.051
	Moderate or severe disability (3-5)	327 (56.2)	46 (68.7)	
Mini-Mental State Examination	No cognitive impairment	312 (63.2)	29 (56.9)	0.336
	Mild cognitive impairment	121 (24.5)	12 (23.5)	
	Severe cognitive impairment	61 (12.3)	10 (19.6)	
Frontal Assessment Battery	Mean (SD)	14 (3.9)	14 (5.1)	0.306
Centre for Epidemiological Studies Depression Scale	Mean (SD)	6.5 (5.5)	7.7 (6.1)	0.124
Discharge to Community Hospital	Yes	139 (23.5)	20 (29.0)	0.315
	No	452 (76.5)	49 (71.0)	
Relationship with caregiver	None	66 (11.2)	6 (8.7)	**0.006**
	Spouse	299 (50.8)	27 (39.1)	
	Child	151 (25.7)	29 (42.0)	
	Sibling	34 (5.8)	7 (10.1)	

	Others	38 (6.5)	0 (0)	

Out of 661 stroke patients recruited at baseline, we included 592 for current analysis. (**Refer Additional File 1**) About 52% of the excluded participants (n = 69) comprised of those who died (n = 36) during the follow up period of one year. Therefore, comparing the baseline characteristics of the included versus excluded sample of stroke patients, we found differences in age, marital status, stroke severity measured on NIHSS and relationship with caregiver, which were statistically significant. The excluded sample mainly comprising of those who died during follow up were older, had higher proportion of severe stroke cases, total dependence on Barthel Index and adult-child caregivers.
